# Initial encounter and discharge disposition of Medicare beneficiaries with post-stroke dysphagia

**DOI:** 10.3389/fstro.2025.1628704

**Published:** 2025-09-02

**Authors:** Molly Jacobs, Richard C. Lindrooth, Marcelo C. Perraillon, Karen Hegland, Robert McGowan, Charles Ellis

**Affiliations:** ^1^Department of Health Services Research, Management and Policy, College of Public Health and Health Professions, University of Florida, Gainesville, FL, United States; ^2^Department of Health Systems, Management & Policy, Colorado School of Public Health, University of Colorado Anschutz Medical Campus, Aurora, CO, United States; ^3^Department of Speech, Language and Hearing Sciences, College of Public Health and Health Professions, University of Florida, Gainesville, FL, United States

**Keywords:** stroke, dysphagia, swallowing, communication, Medicare

## Abstract

**Introduction:**

Dysphagia or disordered swallowing is a post-stroke condition that requires early intervention to improve stroke recovery. Individuals with dysphagia require specialized services to support nutrition and reduce the likelihood of pneumonia after stroke. This study was designed to utilize Medicare claims data to better understand the acute and post-acute pathways of stroke patients with dysphagia.

**Methods:**

Data included 100% of fee-for-service (FFS) Medicare claims for home health agency (HHA), skilled nursing facility (SNF), inpatient, outpatient, and carrier files. The sample included Medicare beneficiaries who incurred an inpatient claim between January 1, 2016, and October 1, 2019, with ischemic or hemorrhagic stroke as the primary diagnosis.

**Results:**

We identified 745,917 unique FFS Medicare beneficiaries with a primary stroke diagnosis; 90% were over age 65. Approximately 79% were non-Hispanic White, 12% were Black/African American, and 6% were Hispanic. Among those identified stroke survivors, 32.5% were diagnosed with dysphagia within 90 days, with slight racial/ethnic variations. Between 68% and 73% of people with dysphagia (PWD) had their initial service encounter in an inpatient hospital facility, 15%−16% in an SNF, and 4%−5% in an outpatient facility, and there was little variation across race/ethnicity. Approximately 10%−12% of PWD were discharged directly home, 32%−36% were discharged to an SNF, and 33%−36% were discharged to a long-term care or rehabilitation facility.

**Discussion:**

In this study of Medicare data, the rate of dysphagia after stroke among Medicare beneficiaries was ~33%. This rate showed only slight variation across racial and ethnic groups. Approximately 70% of PWD were identified in inpatient settings. Only a small percentage (10%) were discharged home, with ~80% moving to facilities offering rehabilitative services (SNF, long-term care, or rehabilitation facility).

## 1 Introduction

Disordered swallowing, or dysphagia, occurs frequently following stroke and can lead to malnutrition, dehydration, aspiration pneumonia, and other health issues during stroke recovery. Primary risk factors for dysphagia after stroke include hypertension, prior stroke, and atrial fibrillation (Song et al., 2024). The current literature suggests that the prevalence of dysphagia after stroke is 42%−46% (Banda et al., 2022; Mao et al., 2024; Song et al., 2024). Among those with dysphagia, the pooled odds of death (OR = 4.7) and pneumonia (OR = 4.08) are four times greater compared to those without dysphagia (Banda et al., 2022). Dysphagia is associated with worse outcomes, reduced independence, and reduced quality of life after stroke (Balcerak et al., 2022). Older adults with dysphagia also experience longer length of stays when hospitalized and substantial costs for their care (Patel et al., 2018). Among those negative outcomes are higher costs of care among individuals with dysphagia after stroke (Bonilha et al., 2014; Muehlemann et al., 2019). Older adults are particularly vulnerable to dysphagia as studies show that up to one-third experience the condition, and a higher prevalence exists in age-related neurological conditions such as Alzheimer's disease and Parkinson's disease (Thiyagalingam et al., 2021).

Clinical practice guidelines for dysphagia after stroke suggest early evaluation and management to prevent the risk of pneumonia and/or death (Choi, 2023; Liu et al., 2018; Sherman et al., 2021; Yang et al., 2023). Consequently, effective evaluation (informal and formal) and interventions are required to facilitate optimal swallowing recovery after stroke (Langton-Frost et al., 2024; Mulheren et al., 2020). Additionally, swallowing ability is critical to secondary stroke prevention as many stroke survivors are required to take multiple medications to reduce their stroke risk (Yu et al., 2024). Many struggle to manage the complications of dysphagia (Helldén et al., 2018). Others are disappointed with the dietary modifications required to manage dysphagia (Helldén et al., 2018). Many stroke survivors also report a lack of support from health care providers to address dysphagia after stroke, particularly when recovery occurs slowly and over time (Helldén et al., 2018).

The timing of early intervention for dysphagia after stroke is critically important (Labeit et al., 2023). Early identification and management are associated with a reduced risk of in-hospital death among stroke patients with malnutrition risk (Zhang et al., 2022). Therefore, timely care is critical to recovery of dysphagia after stroke and a key recommendation of clinical management guidelines for the condition (Liu et al., 2018; Zhang et al., 2022). Delays in screening or comprehensive assessment for dysphagia are associated with increased risk of stroke-related pneumonia (Bray et al., 2017). Delays in dysphagia management overall are also related to longer lengths of stay and greater levels of disability at discharge (Han et al., 2018). Specialized care by individuals who understand the complex mechanisms of swallowing is vitally important to optimizing stroke recovery, reducing mortality and morbidity, and improving quality of life (Labeit et al., 2023).

The presence of dysphagia following stroke plays an important role in discharge disposition post-stroke when detected in the acute phase. For example, stroke patients with dysphagia are significantly more likely to be discharged to institutional settings, such as skilled nursing facilities (SNFs) or long-term care, rather than returning home. Multiple systematic reviews have identified dysphagia as a key clinical factor that, alongside stroke severity, functional impairment, cognitive status, and limited social support, reduces the likelihood of discharge to independent living (Mees et al., 2016; Van der Cruyssen et al., 2014; Burton et al., 2018). However, these reviews were based on studies in which dysphagia was identified in the acute care setting, which may not always be the case, and did not include large cohorts of diverse patients to understand how other demographic factors may impact the pathway of care.

The goal of the current study was to utilize Medicare claims data to better understand the acute and post-acute pathways of diverse patients who experienced stroke and dysphagia. We evaluated the prevalence of dysphagia in a cohort of Medicare beneficiaries who experienced a stroke and identified where the services were initiated in the care continuum and the environments they were discharged to better understand the timeliness of the care settings where care was received. We utilized Medicare claims data obtained from the Centers for Medicare and Medicaid Services (CMS), which are available to researchers for studying health-related conditions, such as dysphagia (Lichtman et al., 2015; Mues et al., 2017). We utilized Medicare data because they offer a large sample of individuals with the condition and allow longitudinal tracking of the services received across health care system units and providers (Lichtman et al., 2015; Mues et al., 2017). This approach allowed the team to explore how and when stroke survivors receive care for dysphagia after stroke and determine which patterns of care are received among a diverse group of individuals with varying sociodemographic characteristics.

## 2 Methods

### 2.1 Institutional review

This study was reviewed and approved by the University of Florida Institutional Review Board as an exempt study, #IRB202300891 due to its use of existing, de-identified data that could not be linked back to individual subjects.

### 2.2 Data source

Data for this project comprised a 100% sample of Medicare claims filed between 2016 and 2019. The Medicare Master Beneficiary Summary File contained information on beneficiary characteristics, such as age, race, sex, and entitlement codes. Claims included outpatient, skilled nursing facility (SNF), home health agency (HHA), inpatient, and carrier files. Outpatient claims included services from hospital outpatient departments, clinics, and ambulatory surgical centers. SNF claims include post-acute care, therapy, rehabilitation, and nursing services. HHA claims covered skilled home health care services, including nursing, physical, occupational, and speech therapy. Inpatient claims included short-term acute care, long-term acute care, inpatient rehabilitation, and inpatient psychiatric care. Carrier files originated from individual practitioners or supplier organizations rather than hospitals or other institutional facilities.

To identify the place of service within inpatient claims, the last four digits of the CMS Certification Number—a unique six-digit identifier assigned by Medicare-certified providers—were used to determine the type of facility (see Appendix). In carrier claims, the place of service (POS) code was used to identify the location where the service was rendered. Each POS code corresponds to a specific type of service location, such as a physician's office (POS 11), inpatient hospital (POS 21), outpatient hospital (POS 22), SNF (POS 31), or home (POS 12). This information was used to classify services across care settings, even when the billing provider was not a facility.

### 2.3 Sample

**Figure 1:**
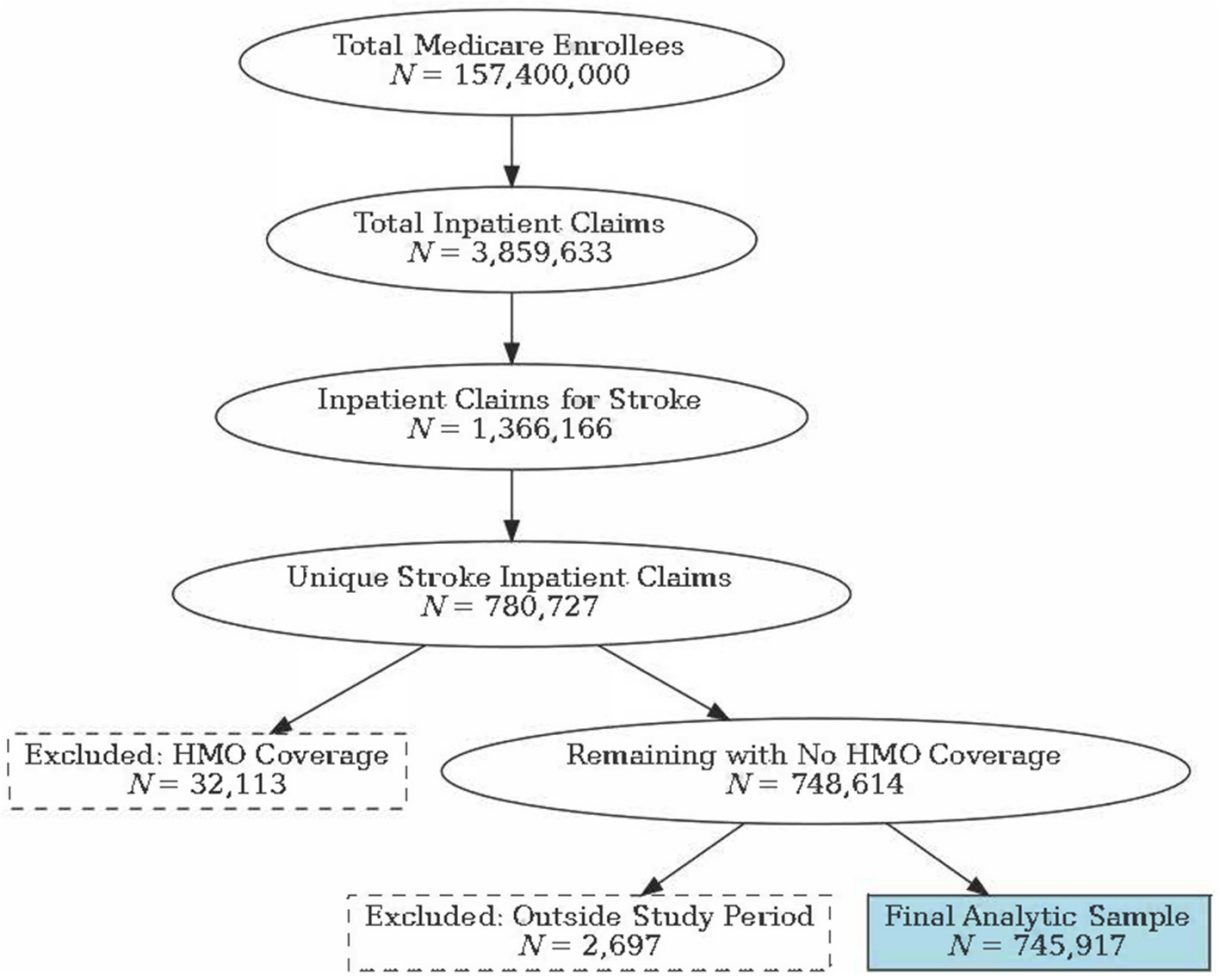
Medicare sample. HMO.

The index hospitalization was defined as an acute care hospital admission for an ischemic or hemorrhagic stroke (see Appendix). The presence of stroke was identified from inpatient claims listing stroke as the primary diagnosis between January 1, 2016, and October 1, 2019. While traditional Medicare operates transparently through a claim-based reimbursement process, allowing all care, providers, and diagnoses to be viewed within the claims, Medicare Advantage (MA) functions under a capitated payment system. Under this system, private insurance companies receive a fixed monthly payment per enrollee to cover all necessary health care services. Because these private insurers manage care internally, they do not submit detailed claims to Medicare. Medicare only collects encounter data from MA plans that summarize the services provided rather than detailing each claim. Therefore, all beneficiaries not enrolled in traditional fee-for-service (FFS) Medicare for 12 months were excluded. This process is illustrated in Figure 1. Post-stroke dysphagia was identified for beneficiaries with a diagnosis code (dysphagia R13.10, R13.11, R13.12, R13.13, R13.14, R13.19, I69.321, I69.921) in the inpatient, outpatient, carrier, HHA, or SNF claim files within 3 months (90 days) of the index hospitalization.

### 2.4 Analysis

To characterize the first-time stroke and dysphagia samples, frequency values for beneficiary-level age, sex, entitlement group, discharge disposition, and location of first dysphagia claim were calculated. Given the proven validity of the Research Triangle Institute (RTI) racial classification, the RTI race variable was used for classification. Entitlement codes included Old Age and Survivors Insurance (OASI), disability insurance benefits (DIB), end-stage renal disease (ESRD), both DIB and ESRD, and beneficiary insured due to Part B immunosuppressive drug. Due to CMS confidentiality restrictions prohibiting reporting cell sizes below 11, DIB, ESRD, and DIB/ESRD were combined into a single category. Because Medicare has more than 40 discharge status codes, facility-level groupings were created. Finally, Charlson Comorbidity Index (CCI) values were calculated for all FFS beneficiaries using diagnoses listed in the outpatient, inpatient, and carrier files following the methodology used by Cenzer et al. (2013) Chi-square tests were used to evaluate differences between racial/ethnic groups. Chi-square test results were considered statistically significant at a *p*-value < 0.05.

## 3 Results

**Figure 2:**
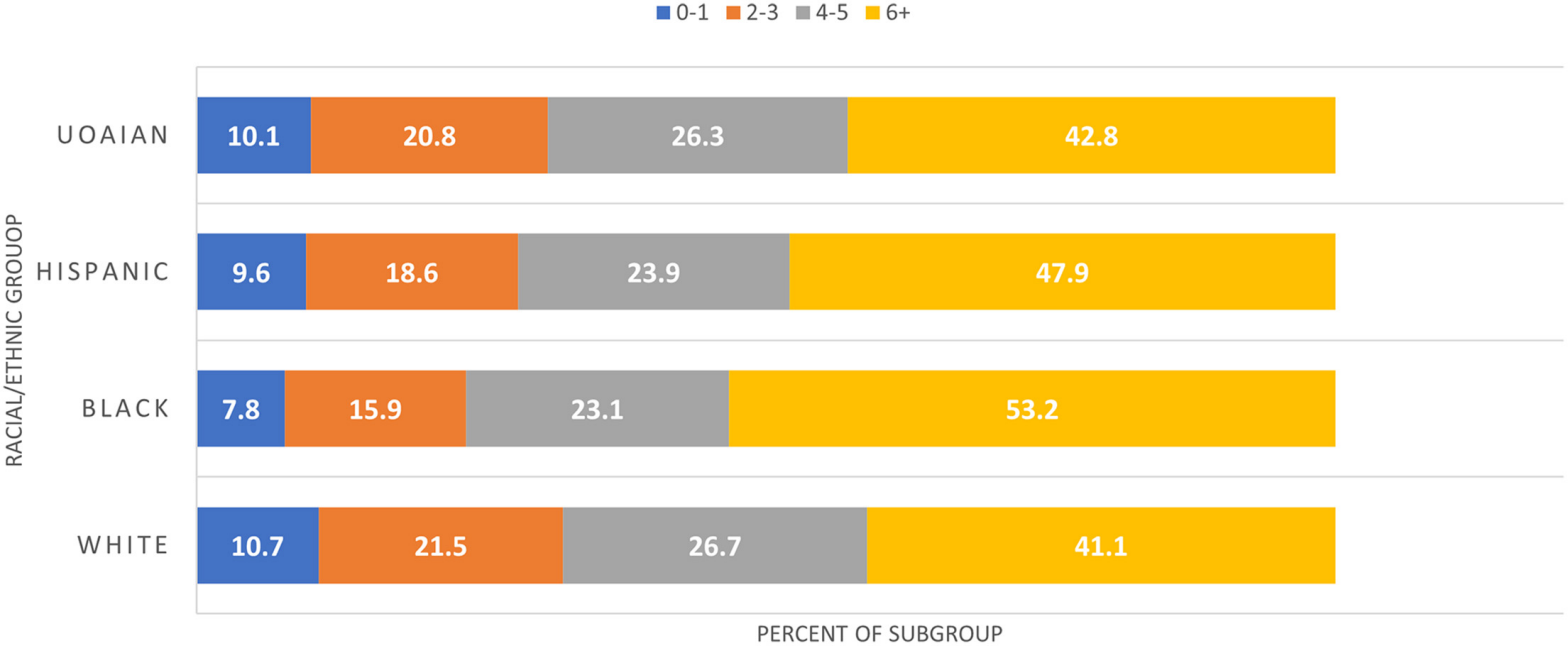
Stroke survivor comorbidities. UOAIAN, Unknown, other, Asian/Pacific Islander, American Indian, and Alaska Native.

The characteristics of the Medicare FFS beneficiaries with a primary stroke diagnosis are listed in Table 1. Most stroke survivors were White (78%), with 12% Black, 6% Hispanic, and 4% unknown, other, Asian/Pacific Islander, American Indian, and Alaska Native (UOAIAN). Approximately 90% of beneficiaries were over 65 and received Medicare through the OASI program, with slight variations between racial and ethnic groups. As seen in Figure 2, Black people with dysphagia (PWD) had the largest portion, with six or more comorbidities (53.2%), followed by Hispanic PWD (47.9%) and UOAIAN PWD (42.8%). Black PWD had the largest portion of females (57%), while UOAIANs were split roughly equally between the sexes. More than 41% of White and 43% UOAIAN stroke survivors had more than six comorbidities on the CCI compared to 53% of Black and 48% of Hispanic stroke survivors. Between 30% and 40% of stroke survivors were discharged directly home, and 17%−20% were discharged to an SNF.

**Table 1:** Characteristics of Medicare fee-for-service stroke survivors.

**Category**	**Group**	**Full sample (*****N*** = **745,917)**		**Non-Hispanic White (*****N*** = **580,972, 77.89%)**		**Black/African American (*****N*** = **89,286, 11.97%)**		**Hispanic (*****N*** = **42,888, 5.75%)**		**Unknown, other, Asian/Pacific Islander, American Indian, Alaska Native (*****N*** = **32,771, 4.39%)**		**Test of between-groups differences**	
		***N***	**%**	***N***	**%**	***N***	**%**	***N***	**%**	***N***	**%**	χ^2^	***p*** **-value**
Age	< 65	67,646	9.07	38,742	6.67	19,330	21.65	6,621	15.44	2,953	9.01		
	≥65	678,271	90.93	542,230	93.33	69,956	78.35	36,267	84.56	29,818	90.99	23308.8	< 0.0001
Sex	Male	340,723	45.68	265,100	45.63	38,758	43.41	20,487	47.77	16,378	49.98		
	Female	405,194	54.32	315,872	54.37	50,528	56.59	22,401	52.23	16,393	50.02	505.46	< 0.0001
Entitlement	OASI	677,815	90.87	541,953	93.28	69,865	78.25	36,208	84.42	29,789	90.9		
	DIB, ESRD, DIB/ESRD, PBID	68,102	9.13	39,019	6.72	19,421	21.75	6,680	15.58	2,982	9.1	23371.83	< 0.0001
Charlson comorbidity index	0–1	76,779	10.29	62,412	10.74	6,946	7.78	4,122	9.61	3,299	10.07		
	2–3	153,566	20.59	124,604	21.45	14,166	15.87	7,980	18.61	6,816	20.8		
	4–5	194,605	26.29	155,103	26.7	20,643	23.12	10,235	23.86	8,624	26.32		
	6+	320,967	43.03	238,853	41.11	47,531	53.23	20,551	47.92	14,032	42.82	5302.74	< 0.0001
Discharge destination	Home	242,068	32.55	191,455	33.05	25,250	28.42	14,863	34.78	10,500	32.1		
	Transfer to acute care	14,289	1.92	10,851	1.87	1,786	2.01	878	2.05	774	2.37		
	SNF	147,911	19.89	115,652	19.96	19,102	21.5	7,218	16.89	5,939	18.16		
	LTC/rehab	144,580	19.44	110,049	19	19,955	22.46	8,011	18.74	6,565	20.07		
	Home health	92,148	12.39	69,615	12.02	12,654	14.24	5,889	13.78	3,990	12.2		
	Died	54,033	7.27	41,567	7.17	5,932	6.68	3,468	8.11	3,066	9.37		
	Hospice/other facility	48,606	6.54	40,147	6.93	4,174	4.7	2,413	5.65	1,872	5.72	2829.93	< 0.0001

OASI, Old Age and Survivors Insurance; DIB, disability insurance benefits; ESRD, end-stage renal disease; PBID, Part B immunosuppressive drug; SNF, skilled nursing facility; LTC, long-term care.

### 3.1 Rate of dysphagia

Characteristics of the Medicare FFS PWD are listed in Table 2. Approximately one-third (32.5%) had a diagnosis of dysphagia. Among those stroke survivors diagnosed with dysphagia, 183,444 (75.8%) were White, 32,792 (13.6%) were Black, 14,264 (5.9%) were Hispanic, and 11,567 (4.8%) were UOAIAN. Most PWD were over 65 years old (94% White, 82% Black, 87% Hispanic, 92% AIAN) and approximately half were female (55% White, 56% Black, 52% Hispanic, and 51% UOAIAN).

**Table 2:** Characteristics of Medicare fee-for-service people with dysphagia.

**Category**	**Group**	**Full sample (*****N*** = **242,067)**		**Non-Hispanic White (*****N*** = **183,444, 75.78%)**		**Black/African American (*****N*** = **32,792, 13.55%)**		**Hispanic (*****N*** = **14,264, 5.89%)**		**Unknown, other, Asian/Pacific Islander, American Indian, Alaska Native (*****N*** = **11,567, 4.78%)**		**Test of between group differences**	
		***N***	**%**	***N***	**%**	***N***	**%**	***N***	**%**	***N***	**%**	***F*** **-stat**	***p*** **-value**
Age	< 65	19,412	8.02	10,673	5.82	6,049	18.45	1,788	12.53	902	7.8		
	≥65	222,655	91.98	172,771	94.18	26,743	81.55	12,477	87.47	10,664	92.2	1.67	0.24
Sex	Male	109,831	45.37	82,790	45.13	14,481	44.16	6,909	48.43	5,651	48.86		
	Female	132,236	54.63	100,654	54.87	18,311	55.84	7,356	51.57	5,915	51.14	0.04	0.85
Entitlement	OASI	222,521	91.93	172,686	94.14	26,713	81.46	12,464	87.37	10,658	92.15		
	DIB, ESRD, DIB/ESRD, PBID	19,546	8.07	10,758	5.86	6,079	18.54	1,801	12.63	908	7.85	1.67	0.24
Charlson comorbidity index	0–1	16,035	6.62	12,881	7.02	1,668	5.09	839	5.88	647	5.59		
	2–3	33,235	13.73	26,742	14.58	3,302	10.07	1,640	11.5	1,551	13.41		
	4–5	62,812	25.95	49,424	26.94	7,013	21.39	3,274	22.95	3,101	26.81		
	6+	129,985	53.7	94,397	51.46	20,809	63.46	8,512	59.67	6,267	54.18	1.03	0.41
Initial service identification	Inpatient facility	101,762	83.22	125,427	68.37	22,789	69.50	9,936	69.66	8,405	72.66		
	Rehabilitation facility	2,386	1.95	7,296	3.98	1,486	4.53	759	5.32	427	3.69		
	Skilled nursing facility	6,389	5.22	29,385	16.02	5,405	16.48	2,192	15.37	1,685	14.57		
	Home health	677	0.55	4,795	2.61	840	2.56	364	2.55	300	2.59		
	Outpatient facility	3,639	2.98	10,541	5.75	1,537	4.69	597	4.19	469	4.05		
	Office	6,104	4.99	4,913	2.68	601	1.83	374	2.62	216	1.87		
	Ambulance/other	1,327	1.08	1,087	0.59	134	0.41	42	0.29	65	0.56	1.83	0.14
Discharge Destination	Home	25,644	10.65	19,902	10.89	2,873	8.83	1,681	11.85	1,208	10.48		
	Transfer to Acute Care	6,572	2.73	4,804	2.63	956	2.94	439	3.09	373	3.24		
	SNF	82,824	34.37	62,643	34.29	11,759	36.13	4,584	32.3	3,838	33.29		
	LTC/Rehab	80,825	33.54	60,427	33.08	11,621	35.71	4,797	33.8	3,980	34.52		
	Home Health	18,255	7.58	13,294	7.28	2,736	8.41	1,271	8.96	954	8.27		
	Died	9,876	4.1	7,675	4.2	1,106	3.4	598	4.21	497	4.31		
	Hospice/Other Facility	16,935	7.03	13,943	7.63	1,491	4.58	821	5.79	680	5.9	1.12	0.39

OASI, Old Age and Survivors Insurance; DIB, disability insurance benefits; ESRD, end-stage renal disease; PBID, Part B immunosuppressive drug; SNF, skilled nursing facility; LTC, long-term care.

### 3.2 Initial service encounter and discharge disposition

**Figure 3:**
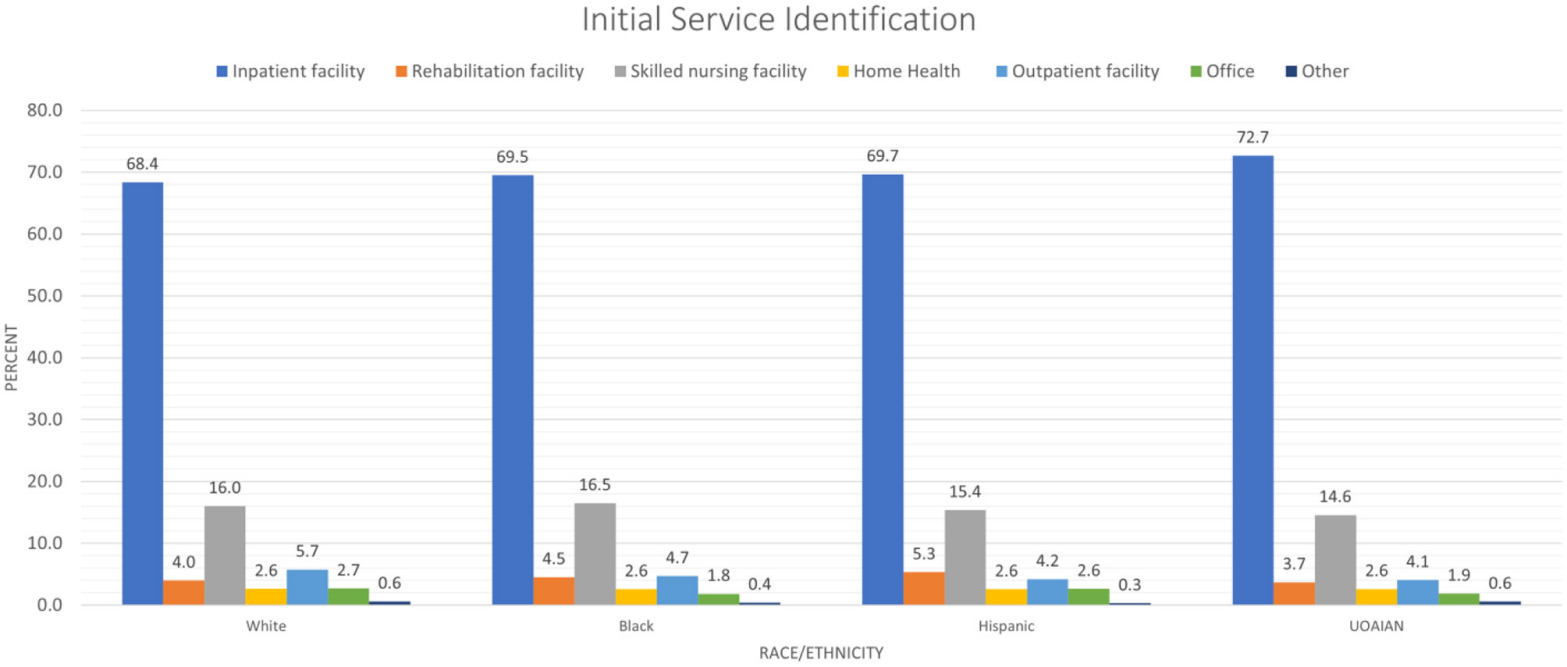
People with dysphagia location of identification. UOAIAN, Unknown, other, Asian/Pacific Islander, American Indian, and Alaska Native.

As seen in Figure 3, between 69% and 73% of PWD had their initial service identified in an inpatient facility, and 15%−17% were identified in an SNF. Approximately 11% of White, 12% of Hispanic, 10% UOAIAN, and 9% of Black PWD were discharged home. However, the majority of PWD were discharged to either an SNF (White 34%, Black 36%, Hispanic 32%, and UOAIAN 33%) or a long-term care/rehabilitation facility (White 33%, Black 36%, Hispanic 34%, and UOAIAN 35%).

## 4 Discussion

In this study of Medicare beneficiaries who experienced a stroke, approximately one-third (32.5%) were treated for dysphagia. It is also notable that ~70% of all individuals with dysphagia experienced their first clinical encounter in an inpatient setting. In contrast, ~10% were discharged home after their inpatient encounter, with the majority (~80%) moving to facilities offering additional rehabilitation services (SNF, long-term care/rehab, HHA). The rate of dysphagia observed in this study is slightly lower than in prior reports. Two prior studies of dysphagia have been completed using Medicare data. In a study of more than 3,000 Medicare beneficiaries in South Carolina, Bonilha et al. (2014) found that 9.9% of stroke survivors exhibited dysphagia. Horn et al. (2022) found a 15.7% rate of dysphagia during inpatient hospitalization in a 2017 Medicare 5% Limited Data Set. Other studies of dysphagia using Medicare data found rates of 45.3% over a 2-year period among Medicare patients with head and neck cancer (Hutcheson et al., 2019) and 4.7% among Medicare patients with metastatic brain cancer (Leiman et al., 2023). Differences in Medicare samples must be considered when evaluating these differences. For example, Bonilha et al. examined Medicare beneficiaries in one state (South Carolina) and excluded individuals under the age of 65. In the current study, we used a national sample that included younger Medicare beneficiaries, representing 5% of the total sample. Therefore, the demographic characteristics of this sample should be carefully considered when interpreting these findings.

The finding of 70% of stroke survivors having their dysphagia identified in an inpatient setting is encouraging when considering that early management stroke guidelines call for dysphagia screening before stroke patients begin eating and drinking for receiving oral medication (Powers et al., 2019). Consequently, managing dysphagia in the early stages of stroke is directly related to proper nutrition and the receipt of medication, which are tied to stroke recovery. More importantly, dysphagia management is critical to reducing the likelihood of stroke survivors being diagnosed with pneumonia. Pneumonia is always a major concern in stroke care as the presence of the condition contributes to incrementally higher cost of care (Katzan et al., 2007) and increased rate of re-hospitalization (Lichtman et al., 2015). At the same time, these findings point to another important issue related to early diagnosis. Approximately one-third of the sample was not identified in inpatient facilities, with roughly 15% being identified in SNFs. This may reflect delays in initial diagnosis and management as care in SNFs occurs after acute inpatient care and, for some, after inpatient rehabilitation care. Further study of this issue by following distinct pathways for individual stroke survivors is required.

The study also showed that < 10% of stroke survivors with dysphagia were discharged home. Therefore, only a small percentage are potentially continuing to exhibit issues with dysphagia in the absence of specialized care. At the same time, roughly 80% were moved from acute care facilities to stroke care facilities offering different levels of rehabilitative care for the condition. Because most PWD after stroke recover within 1 month (Sreedharan et al., 2022), it is tenable that most are receiving the necessary care to minimize the impact of dysphagia on overall stroke recovery.

Understanding the Medicare population, particularly those Medicare beneficiaries experiencing stroke, is critically important. From 2013 to 2019, the number of Medicare hospice beneficiaries with stroke increased more than 50%, yet only half of that increase could be explained by increases in stroke mortality (de Havenon et al., 2024). The study of Medicare beneficiaries using claims data allows the claims of individual beneficiaries to be linked across health care systems, hospitals, rehabilitation units, and providers to determine the type of care received for specific conditions, such as dysphagia. However, there are specific limitations to this work that should be considered. First, Medicare data are designed primarily for billing, not research purposes, and when used for research, the findings can be limited by coding accuracy (Cohen, 2014). Second, the vast majority of Medicare beneficiaries are over the age of 65 (Mues et al., 2017). Third, Medicare data do not include critical behavioral information that contributes to stroke risk or the laboratory tests utilized to manage stroke (Mues et al., 2017). Finally, Medicare data do not capture clinical outcomes for conditions such as dysphagia; thus, we do not have information about the long-term functional impact of the presence of dysphagia in this cohort of stroke survivors.

## 5 Conclusion

In conclusion, this study highlights important patterns in the identification, treatment, and discharge outcomes of dysphagia among Medicare beneficiaries who experienced a stroke. Approximately one-third of stroke survivors were diagnosed with dysphagia, a rate slightly lower than prior reports but still reflective of significant clinical concern given the older age of the Medicare population. The finding that 70% of dysphagia cases were identified during inpatient hospitalization is encouraging and suggests adherence to early stroke management guidelines for dysphagia screening. Furthermore, most patients were referred to rehabilitation facilities, suggesting the need for further research on the quality of treatment in such facilities. Only a small proportion was discharged home directly, which may suggest a need to examine structured care pathways for stroke recovery. While Medicare claims data offer valuable insights by linking care across settings, inherent limitations related to coding accuracy, age restrictions, and a lack of behavioral and laboratory data must be acknowledged. Continued research using complementary data sources is necessary to fully capture the trajectory and management of dysphagia in the stroke population.

## Data Availability

The datasets presented in this article are not readily available because this study utilized the Limited Data Set (LDS) from the Centers for Medicare &amp; Medicaid Services (CMS). CMS makes these files available to researchers as allowed by federal laws and regulations and CMS policy. LDS files contain beneficiary-level health information and are considered identifiable files. Usage of LDS files requires a signed LDS Data Use Agreement (DUA) between CMS and the LDS requester. Requesters are also required to provide a research purpose as part of their request. CMS DUA prohibits all disclosure, sharing, and publication of these data under Section 1106(a) of the Social Security Act [42 U.S.C.§ 1306(a)]. Violators will face criminal penalties under Section 1106(a) of the Social Security Act [42 U.S.C.§ 1306(a)], including a fine not exceeding $10,000 or imprisonment not exceeding 5 years, or both, may apply to disclosures of information that are covered by Section 1106 and that are not authorized by regulation or by Federal law. Requests to access the datasets should be directed to ellisch@phhp.ufl.edu.
