# Supplementary material for: Initial encounter and discharge disposition of Medicare beneficiaries with post-stroke dysphagia
Source: Front Stroke. 2025 Sep 2;4:1628704. doi: 10.3389/fstro.2025.1628704 (PMC12802681; doi:10.3389/fstro.2025.1628704)
Supplement: Supplementary file 1 [file Data_Sheet_1.pdf]

Supplementary Material

Appendix – Codes Used to Identify Stroke

G45.0 G45.1 G45.2 G45.8 G45.9 G46.0 G46.1 G46.2 G46.3 G46.4 G46.5 G46.6 G46.7
G46.8 G97.31

G97.32 I60.00 I60.01 I60.02 I60.10 I60.11 I60.12 I60.20 I60.21 I60.22 I60.30 I60.31 I60.32
I60.4 I60.50

I60.51 I60.52 I60.6 I60.7 I60.8 I60.9 I61.0 I61.1 I61.2 I61.3 I61.4 I61.5 I61.6
I61.8 I61.9 I63.00 I63.011

I63.012 I63.013 I63.019 I63.02 I63.031 I63.032 I63.039 I63.09
I63.10 I63.111 I63.112 I63.113 I63.119

I63.12 I63.131 I63.132 I63.133 I63.139 I63.19 I63.20 I63.211
I63.212 I63.213 I63.219 I63.22 I63.231

I63.232 I63.233 I63.239 I63.29 I63.30 I63.311 I63.312 I63.313
I63.319 I63.321 I63.322 I63.323 I63.329

I63.331 I63.332 I63.333 I63.339 I63.341 I63.342 I63.343
I63.349 I63.39 I63.40 I63.411 I63.412 I63.413

I63.419 I63.421 I63.422 I63.423 I63.429 I63.431 I63.432
I63.433 I63.439 I63.441 I63.442 I63.443

I63.449 I63.49 I63.50 I63.511 I63.512 I63.513 I63.519 I63.521
I63.522 I63.523 I63.529 I63.531 I63.532

I63.533 I63.539 I63.541 I63.542 I63.543 I63.549 I63.59 I63.6
I63.8 I63.81 I63.89 I63.9 I66.01 I66.02

I66.03 I66.09 I66.11 I66.12 I66.13 I66.19 I66.21 I66.22 I66.23 I66.29 I66.3 I66.8 I66.9
I67.841 I67.848

I67.89 I97.810 I97.811 I97.820 I97.821
